# Supplementary material for: Diversity of Ophiostomatoid Fungi Associated with Dendroctonus armandi Infesting Pinus armandii in Western China
Source: J Fungi (Basel). 2022 Feb 22;8(3):214. doi: 10.3390/jof8030214 (PMC8951329; doi:10.3390/jof8030214)
Supplement: Supplementary file 1 [file jof-08-00214-s001.zip › jof-1598854-supplementary.pdf]

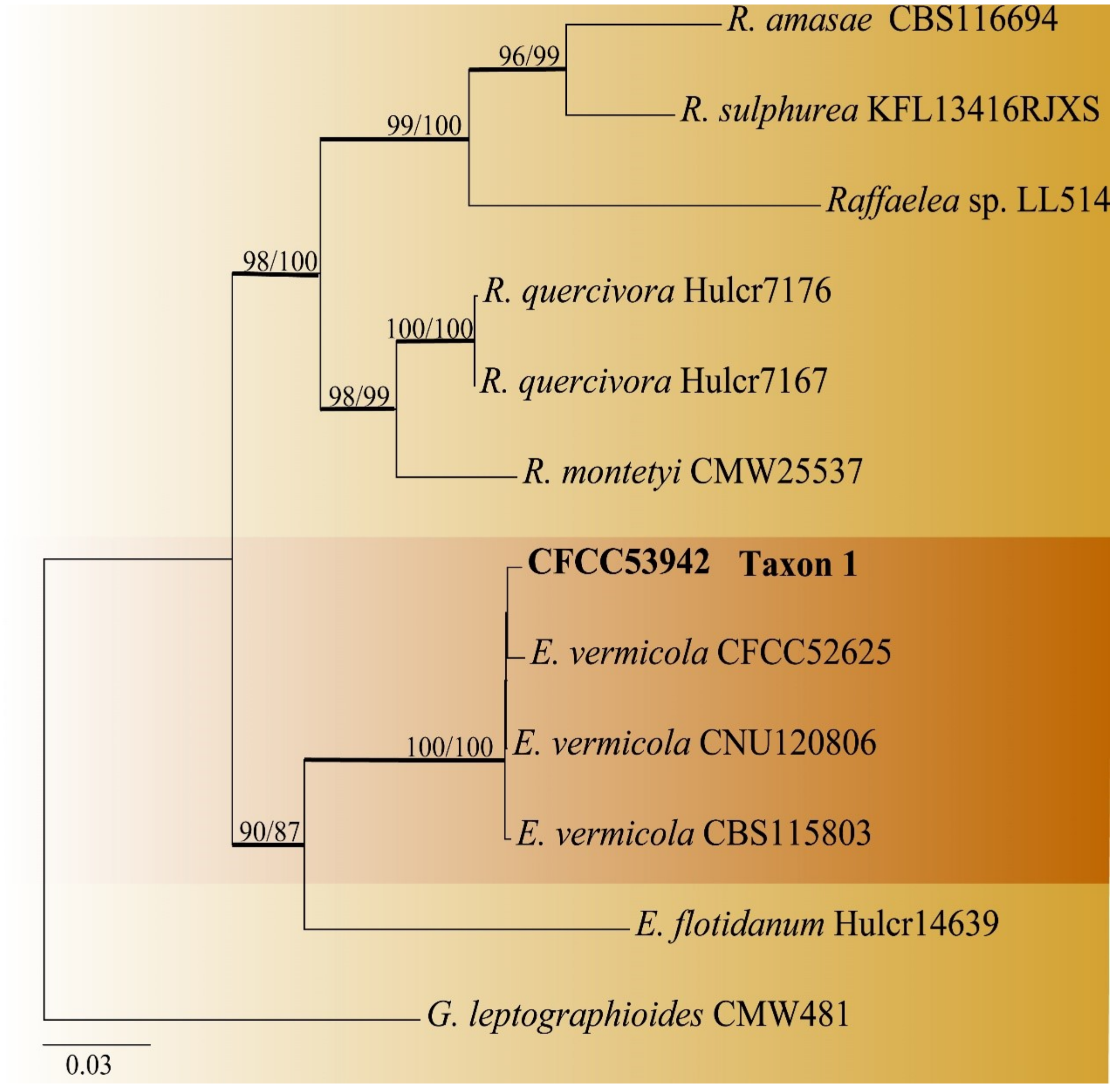

Supplementary Figure S1. ML tree of *Esteya* and related taxa (Taxon 1) generated from the combined (LSU+TUB2) sequence data. Novel sequences obtained in this study are presented in bold typeface. Bold branches indicate posterior probability values ≥ 0.9. Bootstrap values ≥ 70% for ML and MP are indicated above branches.

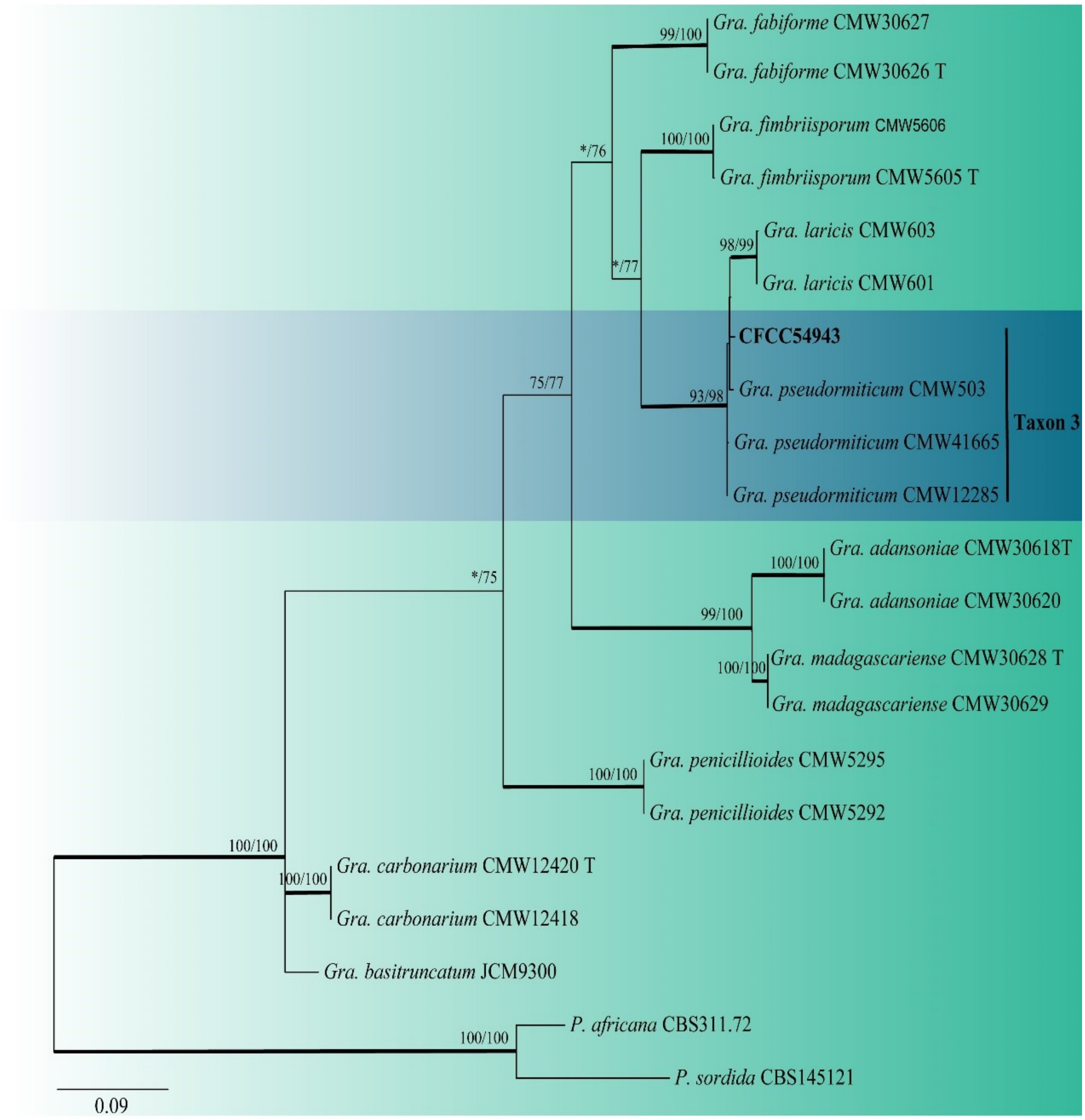

Supplementary Figure S2. ML tree of *Graphium* (Taxon 3) generated from the combined (ITS+tEF1-α) sequence data. Novel sequences obtained in this study are presented in bold typeface. Bold branches indicate posterior probability values ≥ 0.9. Bootstrap values ≥ 70% for ML and MP are indicated above branches. Bootstrap values < 70% are indicated by *.
